# Validation of Multidimensional Scale of Perceived Social Support (MSPSS) in Vietnamese Among People Living with HIV/AIDS

**DOI:** 10.1007/s10461-022-03974-1

**Published:** 2023-01-09

**Authors:** Pham Tieu Kieu, Nguyen Lam Vuong, Do Van Dung

**Affiliations:** 1 Faculty of Public Health, University of Medicine and Pharmacy at Ho Chi Minh City, 217 Hong Bang Street, Ward 11, District 5, Ho Chi Minh City, Vietnam; 2 Oxford University Clinical Research Unit (OUCRU), 764 Vo Van Kiet Street, Ward 1, District 5, Ho Chi Minh City, Vietnam

**Keywords:** HIV/AIDS, Multidimensional Perceived Social Support Scale, MSPSS in Vietnamese, Reliability, Validity

## Abstract

Social support plays a vital role in the health of HIV/AIDS patients, but there needs to be a validated instrument to measure social support in Vietnam. This cross-sectional study was to validate a Vietnamese translation of the Multidimensional Perceived Social Support Scale (MSPSS). The study had three stages: [1] translation to Vietnamese, [2] pilot testing, and [3] validation of the translation. Stage 1, including forward and backward translation by four independent translators, resulted in a good content validity translation. Pilot testing was done on 30 HIV/AIDS patients: the translation was understandable, and no change was required. Five hundred HIV/AIDS patients were recruited in stage 3. The translation had excellent internal consistency (Cronbach’s alpha: 0.90), good test-retest reliability (intra-class correlation coefficient: 0.95), and good concurrent validity. Construct validity was well established by confirmatory factor analysis. The Vietnamese translation of the MSPSS is reliable and valuable for measuring perceived social support.

## Introduction

With the development and availability of anti-retroviral treatment (ART), human immunodeficiency virus (HIV) infection and acquired immunodeficiency syndrome (AIDS) have become manageable chronic diseases [1]. However, HIV/AIDS is still one of the top 10 threats to global health, according to the World Health Organization in 2019 [2]. HIV/AIDS damages infected patients not only in physical health but also in psychological health due to social discrimination. People living with HIV/AIDS suffer from interpersonal relationships and social integration difficulties. Particularly for patients in the early phase without apparent symptoms, the psychological impact is much more significant than physical injury. Psychological trauma also affects the treatment compliance of patients and has a negative impact on physical health. Therefore, social support plays a vital role in both the physical and psychological health of HIV/AIDS patients [3]. Social support helps to prevent HIV-related risk behaviors and to promote healthy behaviors and treatment adherence. In contrast, lack of social support is associated with physical and mental impairment and a faster progression from HIV infection to AIDS [4]. In general, high levels of social support can improve physical and psychological health, reducing mortality and improving the quality of life (QOL) of patients with HIV/AIDS [5].

Social support is a multidimensional construct, which is conceptualized as having three dimensions: (i) structural dimension (represented by social support networks), (ii) functional dimension (represented by received social support exchanges), and (iii) perceptual dimension (represented by appraisals of perceived social support) [6]. Social support can be defined as the amount of assistance one gets through interactions with other people. The support can be either emotional (e.g., empathy), tangible (e.g., practical help), or informational [7]. Perceived social support is the most pronounced influence on psychological health and well-being [8]. A simple way to assess psychological health is through self-reported instruments. Various tools have been developed to assess psychological health and social support. However, there are few tools specific for perceived social support, particularly for HIV/AIDS patients. The Multidimensional Scale of Perceived Social Support (MSPSS) assesses perceived social support in various populations. This self-rating instrument measures perceived social support from three sources: family, friends, and significant others. It has been translated into more than 35 languages. Its reliability and validity have been widely demonstrated in various groups, including students, teachers, pregnant women, adolescents, the elderly, and psychiatric patients [9–19]. However, the MSPSS has not been widely used in HIV/AIDS patients [11].

In Vietnam, approximately 215,000 people are living with HIV/AIDS as of December 2020. The number of newly diagnosed HIV infections is around 14,000 yearly [20]. Social support is essential in managing HIV/AIDS, but there is still no validated instrument for use in Vietnam. Some studies in Vietnam have used the Vietnamese translation of MSPSS for different groups [21–23]. However, there is still no formal validation of the Vietnamese translation of MSPSS. Also, English is not an official language in Vietnam. Therefore, a formal validation of the Vietnamese translation of MSPSS is required. This study was conducted to translate the MSPSS instrument to Vietnamese and validate it in HIV/AIDS patients. With this study’s results, physicians and investigators will have a reliable instrument to assess the perceived social support of HIV/AIDS patients in Vietnam. Using this available instrument can also help researchers reduce workload while ensuring the quality of the tool.

## Methods

This cross-sectional study was conducted from January to March 2021 in the HIV/AIDS Outpatient Department of Medical Center District 8, a primary healthcare center in Ho Chi Minh city, Vietnam. Ho Chi Minh City has the highest number of HIV/AIDS patients, accounting for a quarter of the total number of cases in the country. This medical center was chosen as the study site because it is one of the most long-standing HIV/AIDS management centers with the highest number of patients in Ho Chi Minh city. The characteristics of patients in this center are representative of people living with HIV/AIDS across the country [20].

The study had three stages: [1] translation to Vietnamese and evaluation of content validity, [2] pilot testing, and [3] reliability and validity evaluation of the Vietnamese translation of the MSPSS. In stages 2 and 3, patients treated at the study site were conveniently sampled. Inclusion criteria were: (i) 18 years of age or older, (ii) confirmed diagnosis with HIV/AIDS, (iii) ability to communicate in the Vietnamese language, and (iv) agreed to participate in the study by written informed consent. Exclusion criteria were: (i) a diagnosis of mental disorders and (ii) participation in stage 2 (for stage 3 only).

The study was approved by the Ethics Committee of the University of Medicine and Pharmacy at Ho Chi Minh City, Vietnam (Number 937/HDĐĐ-ĐHYD, dated 14th December 2020). We got the agreement from the author of the MSPSS instrument via email for the translation. All participants were explained and fully understood the study’s objectives, procedures, benefits, and risks prior to providing informed consent. Participants were also informed that they could withdraw from the study at any time without any penalty or impact on their management at the center.

### Instruments

Three instruments were used in this study: (i) the MSPSS, (ii) Medical Outcomes Study: Social Support Survey (MOS-SSS), and (iii) Center for Epidemiologic Studies Depression Scale (CES-D).

The MSPSS is a 12-item self-reported instrument measuring perceived social support from three sources: family, friends, and significant others. Participants rated the MSPSS items using a seven-point Likert scale ranging from 1 (strongly disagree) to 7 (strongly agree). Each dimension (family, friends, significant others) has four items. Sub-dimensional scores are the mean score of the corresponding four items. The overall MSPSS score is the mean score of all 12 items. Higher scores indicate higher levels of perceived social support. The MSPSS has high internal consistency reliability with Cronbach’s alpha of 0.85–0.91 and good test-retest reliability. Construct validity and concurrent validity have also been established [11].

The MOS-SSS is a 19-item self-administered instrument measuring the functional aspects of perceived social support (tangible support, emotional-informational support, positive social interactions, and affectionate support) and one additional item assessing the number of other close relatives and friends. A five-point Likert rating scale from 1 (none of the time) to 5 (most of the time) is used. The overall MOS-SSS score is the mean of all 19 items. Similar to the MSPSS, higher scores indicate higher levels of social support. The MOS-SSS has good internal consistency reliability with Cronbach’s alpha of 0.95–0.97. This tool has been translated, and the Vietnamese translation has been validated [24].

The CES-D is a 20-item self-reported scale that measures depressive symptoms severity in the general population. Each item is rated from 0 (rarely or none of the time) to 3 (most or almost all the time). A score of positive items is reversed. The overall CES-D score is the total score of all 20 items and ranges from 0 to 60. Higher scores indicate greater depressive symptoms. The CES-D has good internal consistency reliability, which is reliable and valuable for screening depressive symptoms in HIV outpatient clinics in Vietnam [25].

### Stage 1: Translation to Vietnamese

We used a translation procedure proposed by Guillemin et al. [26] to translate the MSPSS into Vietnamese. This stage contained five steps: (i) forward and backward translation, (ii) synthesizing, (iii) evaluation by an expert panel, (iv) content validity assessment, and (v) pilot study. The original English version was independently translated into Vietnamese by two translators with good English proficiency, one was a healthcare professional, and another was not a healthcare professional. Each translation version was then independently back-translated into English by two translators who had never known the MSPSS and perceived social support. Next, all four translators discussed and synthesized the pre-final Vietnamese translation. This version was then reviewed and edited by six experts on Substance Addiction - HIV/AIDS, Public Health, Psychology, and Translation. The experts assessed the content validity based on the relevance and clarity of each sentence and came up with the final version. This version was again back-translated into English by another expert interpreter to ensure that it was equivalent to the original version.

### Stage 2: Pilot Study

A sample size of 30 to 40 participants is considered appropriate in a pilot study [26]. The Vietnamese translation was pilot tested among 30 HIV/AIDS patients. These patients were not included in stage 3. This translation was easy to understand by all participants. No changes were made to the translated version after the pilot study.

### Stage 3: Reliability and Validity Evaluation of the Vietnamese Translation of MSPSS

The minimum sample size for the CFA method and Cronbach’s alpha test has yet to reach a consensus. Previous studies were mainly based on experience, with the minimum recommended sample size of 200–500. This sample size was considered suitable for factor analysis, meeting the criteria of 3 to 10 samples for one variable, and also suitable with many other hypotheses about sample size to assess the model’s fit [27]. A rule of thumb for a factor analysis study’s sample size is as follows: 200 is acceptable, 300 is good, and 500 is very good [28]. In this study, we selected a sample size of 500 as it was sufficient for the factor analysis to confirm the structure of the MSPSS.

In this stage, 500 eligible HIV/AIDS patients were enrolled to complete a face-to-face interview using a questionnaire with four parts: demographic characteristics, the MSPSS, MOS-SSS, and CES-D. The MOS-SSS and CES-D instruments were selected to evaluate the concurrent validity of the MSPSS. To evaluate test-retest reliability, 131 patients completed the Vietnamese translation of MSPSS again four weeks after the first interview.

### Statistical Analysis

The internal consistency reliability of the MSPSS was measured by Cronbach’s alpha and item-total correlation coefficients. Cronbach’s alpha measures the extent to which the items consistently measure the same thing, with a value of ≥ 0.80 indicating good internal consistency [29]. The item-total correlation indicates whether the response of every item is consistent with the average behavior of the scale. Higher values indicate better consistency but a correlation coefficient of ≥ 0.30 is acceptable [29]. The test-retest reliability was evaluated using the intraclass correlation coefficient (ICC). An ICC value of > 0.75 indicates good test-retest reliability [30].

Construct validity was evaluated using confirmatory factor analysis (CFA) based on the established three-factor model. The Chi-squared statistic was used to identify whether the model fits the data well. However, the Chi-squared is normally inflated by the large sample size and thus rejects the model. Comparative Fit Index (CFI), Tucker-Lewis Index (TLI), Standardized Root Mean Square Residual (SRMR), and Root Mean Square Error of Approximation (RMSEA) were used to assess the goodness of fit of the model. Values of CFI and TLI of > 0.9 indicate a well-fitting model [31]. The SRMR and RMSEA indicate a ‘badness of fit’ or ‘lack of fit’; thus, smaller values indicate a closer fit between the model and the data. The values of SRMR and RMSEA of < 0.08 indicate a good fit [32]. The concurrent validity of the MSPSS was examined using Spearman’s correlation coefficients with the MOS-SSS and CES-D. All analyses were done using Stata version 14.2.

## Results

### Translation to Vietnamese and Pilot Testing

The translations in both the forward and backward translations had some differences in wording and expression but not much difference in meaning. The translations were compared between each translation and compared with the original version. The translations were discussed thoroughly by the four translators to come up with a unified translation. According to experts’ comments, the translation was close to the original version, which accurately presented the original version’s content.

The final translation had good content validity and was usable in the pilot testing stage. There was a proposal to use a 5-point Likert scale instead of a 7-point scale to make it easier to answer. However, most participants thought the 7-point Likert scale was relatively easy to answer. Therefore, the 7-point Likert scale remains the same as the original version. Most participants (28/30 patients, 93%) thought that the scale was easy to understand and answer, with no sentences confusing or confusing the meaning of the question. No suggestions for editing words or expressions; thus, no change was made after this stage.

### Baseline Characteristics of Participants in Stage 3

Among 500 participants in stage 3 of the study, the mean age was 36.9 ± 8.5 years, and men were predominant (74.2%) (Table 1). Most participants were single (48.8%) or married (36.4%), were employed (72.6%), and were living with their family (78.4%) when participating in the study. Most participants (77.2%) had told others about their HIV status, but only 10.2% were participating in a peer group. The median (25th ; 75th percentiles) duration of HIV infection and ART use was 7.6 (2.7; 13.1) and 5.7 (2.6; 11.2) years, respectively. The baseline characteristics of 131 participants who participated in the second interview were similar to those of all participants.

**Table 1:** Baseline characteristics of participants

	All participants (N = 500)	Participants with 2 interviews (N = 131)
Age (years)	36.9 ± 8.5 (19; 67)	34.8 ± 7.9 (19; 58)
Gender male	371 (74.2)	100 (76.3)
Marital status		
Single	244 (48.8)	68 (51.9)
Married	182 (36.4)	42 (32.1)
Separated or divorced	33 (6.6)	9 (6.8)
Live-in partner	32 (6.4)	11 (8.4)
Widowed	9 (1.8)	1 (0.8)
Living with		
Family	392 (78.4)	102 (77.9)
Other people	39 (7.8)	11 (8.4)
Alone	69 (13.8)	18 (13.7)
Educational level		
Under primary school	42 (8.4)	16 (12.2)
Primary school	55 (11.0)	12 (9.2)
Secondary school	171 (34.2)	40 (30.5)
High school	141 (28.2)	39 (29.8)
Higher education	91 (18.2)	24 (18.3)
Employment status		
Employed	363 (72.6)	97 (74.0)
Housewife or unemployed	79 (15.8)	22 (16.8)
Unable to work or retired	14 (2.8)	3 (2.3)
Other	44 (8.8)	9 (6.9)
Telling others about HIV status (yes)	386 (77.2)	106 (80.9)
Participating in a peer-group (yes)	51 (10.2)	12 (9.1)
Duration of HIV infection (years)	7.6 (2.7; 13.1)	4.1 (2.5; 10.3)
Duration of ART regiments (years)	5.7 (2.6; 11.2)	3.6 (2.4; 8.8)

Statistics are mean ± standard deviation (min; max) or median (25th ; 75th percentiles) for continuous variables and n (%) for categorical variables

HIV, human immunodeficiency virus; ART, anti-retroviral treatment

### Reliability of the Vietnamese Translation of the MSPSS

The mean overall MSPSS score was 4.97 ± 1.39 among 500 participants at the first interview, which indicated a relatively good perceived social support in the study population. The ‘family’ and ‘significant others’ subscales had higher mean scores than the ‘friends’ subscale (Table 2). The Vietnamese translation of the MSPSS had good internal consistency with Cronbach’s alpha of 0.90 for the overall scale and from 0.89 to 0.92 for the three subscales. The item-total correlation coefficients ranged from 0.65 to 0.74, which also showed that all the questions in the scale were not redundant or had no duplicate content. The test-retest reliability was good, with ICCs of 0.954 for the overall scale and from 0.945 to 0.971 for the three subscales when re-interviewing 131 participants four weeks after the first interview.

**Table 2:** Reliability of the MSPSS and its subscales

Item number and description	Mean ± SD	Item-total correlation	Cronbach’s alpha	ICC (95% CI)*
**Family subscale**	5.30 ± 1.73		0.89	0.945 (0.929; 0.958)
Q3. My family really tries to help me.	5.57 ± 1.91	0.70		
Q4. I get the emotional help and support I need from my family.	5.37 ± 1.92	0.74		
Q8. I can talk about my problems with my family.	4.82 ± 2.20	0.67		
Q11. My family is willing to help me make decisions.	5.42 ± 1.91	0.69		
**Friends subscale**	4.04 ± 1.94		0.92	0.965 (0.955; 0.973)
Q6. My friends really try to help me.	4.18 ± 2.16	0.69		
Q7. I can count on my friends when things go wrong.	3.92 ± 2.17	0.67		
Q9. I have friends with whom I can share my joys and sorrows.	4.38 ± 2.15	0.67		
Q12. I can talk about my problems with my friends.	3.68 ± 2.18	0.65		
**Significant others subscale**	5.59 ± 1.70		0.92	0.971 (0.963; 0.978)
Q1. There is a special person who is around when I am in need.	5.63 ± 1.91	0.66		
Q2. There is a special person with whom I can share my joys and sorrows.	5.52 ± 1.93	0.72		
Q5. I have a special person who is a real source of comfort to me.	5.65 ± 1.86	0.73		
Q10. There is a special person in my life who cares about my feelings.	5.54 ± 1.88	0.70		
**MSPSS total**	4.97 ± 1.39		0.90	0.954 (0.941; 0.964)

*ICC was analyzed among 131 participants to evaluate the test-retest reliability

CI, confidence interval; ICC, intraclass correlation coefficient; MSPSS, Multidimensional Perceived Social Support Scale; SD, standard deviation

### Construct Validity of the Vietnamese Translation of the MSPSS

**Fig. 1:**
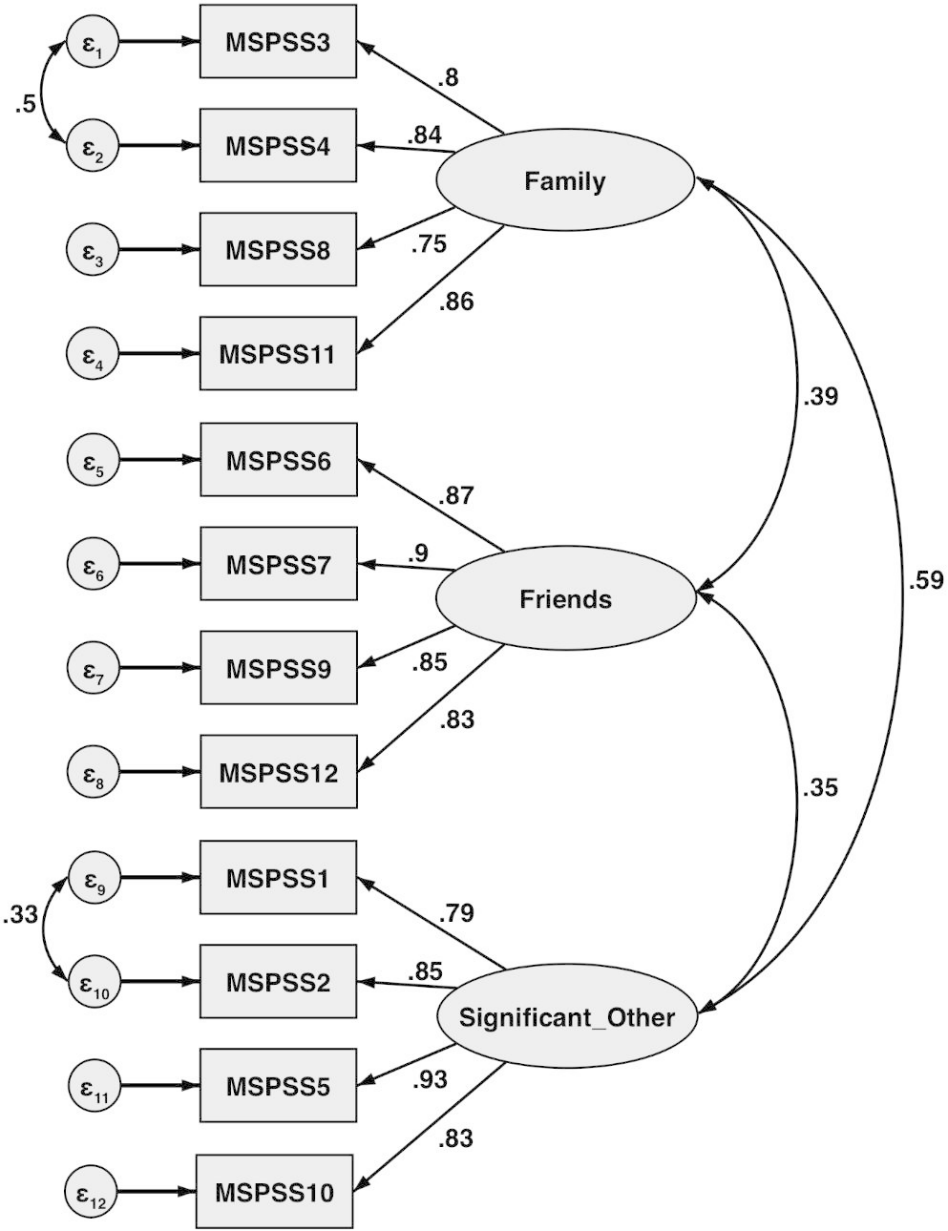
Factor structure of the Vietnamese translation of the MSPSS. (MSPSS, Multidimensional Scale of Perceived Social Support)

Confirmatory factor analysis was performed to test the three-factor structure and compare it with the two-factor structure of the MSPSS. Although the Chi-square test showed a significant difference between the original three-factor model and the expected model (Chi-square = 164.77, p < 0.001), all other parameters (RMSEA: 0.069; SRMR: 0.03; CFI: 0.975; and TLI: 0.966) revealed a good fit for the model specified. The three-factor model was better than the two-factor model in all indices, including lower RMSEA and SRMR, higher CFI and TLI, and lower Akaike Information Criterion (AIC) (Table 3). Standardized factor loadings ranged from 0.75 to 0.93, indicating that the factors provided a good explanation of the variation in the items. The correlation between ‘family’ and ‘significant others’ was quite strong (0.59), whereas the correlation between the other pairs was moderate (0.39 between ‘family’ and ‘friends’, and 0.35 between ‘friends’ and ‘significant others’) (Fig. 1).

**Table 3:** Fit indices for three-factor and two-factor models

	Three-factor model	Two-factor model
Statistic value (chi-square)	164.77	1107.52
Degree of freedom	115	119
P value	< 0.001	< 0.001
RMSEA (90% CI)	0.069 (0.057; 0.080)	0.199 (0.189; 0.210)
SRMR	0.030	0.113
CFI	0.975	0.773
TLI	0.966	0.717
AIC	20928.89	21863.64

AIC, Akaike information criterion; CFI, Comparative fit index; CI, confidence interval; RMSEA, Root mean square error of approximation; SRMR, Standardized root mean square residual; TLI, Tucker-Lewis index

### Concurrent Validity of the Vietnamese Translation of the MSPSS

**Fig. 2:**
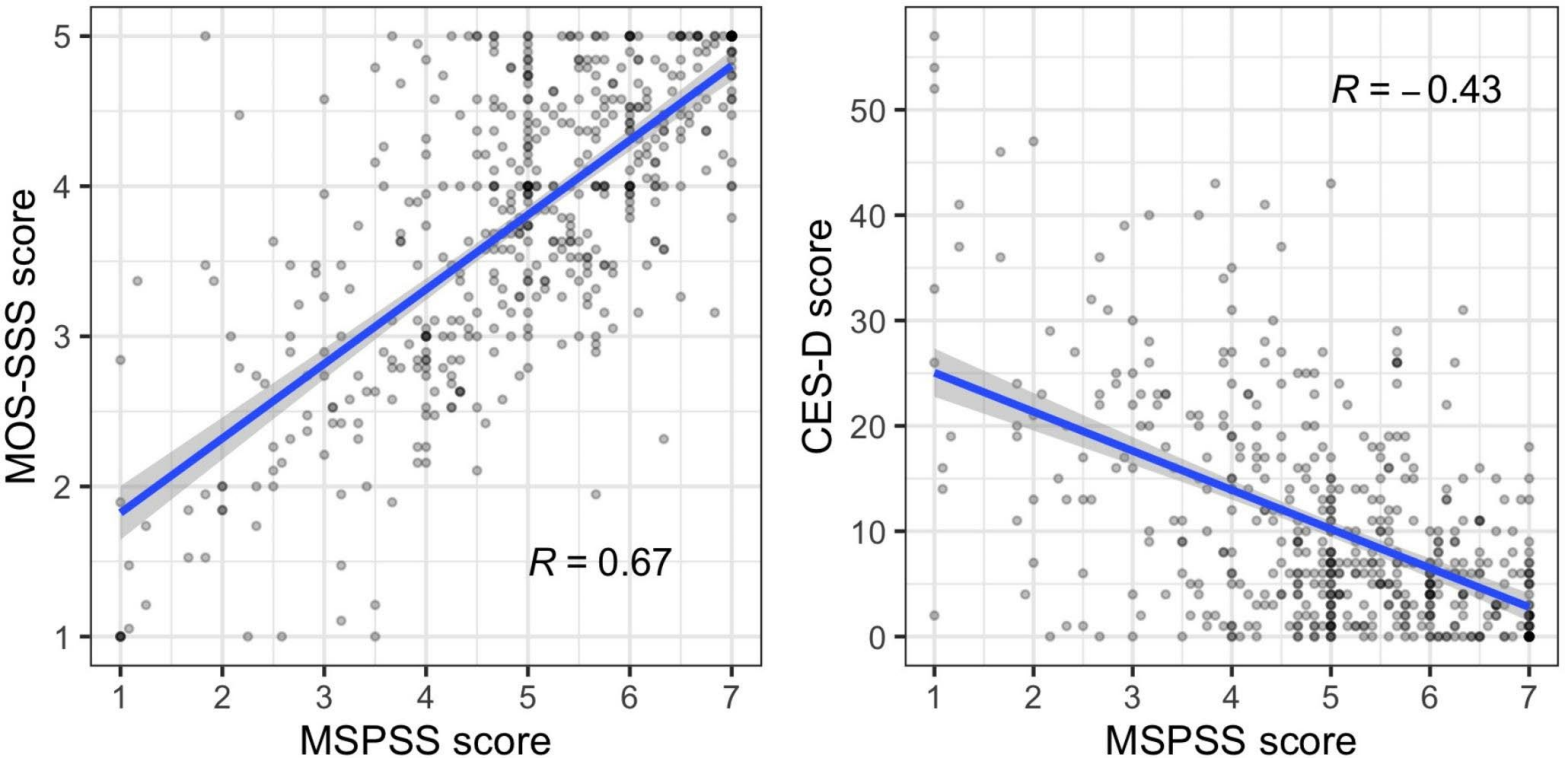
Correlation between the MSPSS with MOS-SSS and CES-D. (Each grey point represents each individual participant. Blue lines are the regression line from linear regression models and grey regions are the 95% confidence interval. The number inside each scatter plot represents the Spearman’s rank correlation coefficient. CES-D, Center for Epidemiologic Studies Depression Scale; MOS-SSS, Medical Outcomes Study: Social Support Survey; MSPSS, Multidimensional Scale of Perceived Social Support)

The overall MSPSS score was positively correlated with the MOS-SSS score (Spearman’s correlation coefficient: 0.67) and was negatively correlated with the CES-D score (Spearman’s correlation coefficient: -0.43). These results were as expected since the MOS-SSS measures the functional aspects of perceived social support, and the CES-D measures depressive symptoms severity (Fig. 2). Higher perceived social support from the three sources (i.e., higher MSPSS score) was associated with higher functional aspects of perceived social support (i.e., higher MOS-SSS score) and lower depressive symptoms severity (i.e., lower CES-D score). These findings confirmed a good concurrent validity of the Vietnamese translation of the MSPSS.

## Discussion

Social support can promote healthy behaviors and treatment adherence and improve the QOL of HIV/AIDS patients. Although social support plays an essential role in preventing HIV-related risk behaviors, there was no linguistically relevant instrument for measuring social support among this population in Vietnam before this study was conducted. Our results indicate that the Vietnamese translation of the MSPSS is a reliable and valid instrument to assess perceived social support for HIV/AIDS patients in Vietnam.

In stages 1 and 2 of this study, we formally translated the original MSPSS questionnaire into Vietnamese. These stages are essential in the translation procedure as recommended by Guillemin et al. [26]. In practice, research questionnaires are not always translated appropriately before using in new temporal, cultural, or linguistic settings, which leads to a risk that the translations may not accurately reflect what they are supposed to measure in the original questionnaire. Instruments should be translated into a specific language of the targeted population and be pretested after that [26]. As mentioned in the introduction, some studies used the Vietnamese translation of the MSPSS in other contexts [21–23]. However, the translation and validation were not properly performed as recommended. In our study, the translation was done forward and backward by four independent translators, including both healthcare professionals and non-healthcare professionals, to ensure the validity and reliability of the translation. Results from stages 1 and 2 show that the translation is appropriately worded and captures the meaning of the original MSPSS instrument. This translation can be used not only for HIV/AIDS people but also for all other people in Vietnam.

The MSPSS has excellent internal consistency and excellent test-retest stability. This result was similar to that reported for the original version and many other studies worldwide [12, 16, 17, 33, 34]. The stability of the MSPSS over four weeks was very good, with ICCs of more than 0.9. Our study’s ICCs were higher than those in the original study (ICC = 0.85) over 8–12 weeks [34]. The difference may be due to the shorter interval between the two interviews in our study. Other studies also showed a good test-retest reliability of the MSPSS in various populations and countries [14, 16, 34, 35].

In terms of construct validity, although the Chi-squared test indicated that the model did not fit the data well, the other fit indices, including CFI, TLI, SRMR, and RMSEA, revealed that the three-factor model of MSPSS was a good fit. Many validation studies also reported that the Chi-squared test was unsatisfactory with the three-factor model, but other fit indices showed a good fit [12, 17, 19, 33, 35]. Our factorial analysis also confirmed that the three-factor model showed a better fit than the two-factor model. This result is consistent with the original solution of Zimet et al. from 1988 [34] and other subsequent studies in different populations from many countries [12, 35–37]. Our study confirms the previous evidence that the MSPSS is valid to assess perceived social support differentiating the three sources ‘family’, ‘friends’, and ‘significant others’. Furthermore, the correlation between the three factors and the standardized factor loading coefficient is higher than the threshold. The highest correlation was between ‘family’ and ‘significant others’ factors, putting a problem that these factors might be indistinguishable. This result suits Vietnamese culture, where people usually live in a big family and are close to family members. As expected, the MSPSS had excellent concurrent validity with a positive correlation with the MOS-SSS and a negative correlation with the CES-D. These findings are similar to previous studies [16, 17].

Social support highly affects treatment adherence and enhances the QOL of HIV/AIDS patients [38]. Social support measurement yields important information in health care and treatment for HIV/AIDS patients. To our knowledge, this is the first study that evaluated the reliability and validity of the MSPSS in the HIV/AIDS population. There is an urgent need for research tools with specific cultures and languages for each country and region, particularly in low-to-middle-income settings, to improve the quality of studies [39, 40]. Researchers and health professionals can use the Vietnamese translation of MSPSS or the combination of the MSPSS and MOS-SSS as screening instruments for routine clinical care for HIV/AIDS patients. The MSPSS is simple and has a high level of reliability and validity. Such applications can help alleviate the lack of information about social support and improve the QOL in this vulnerable population in Vietnam and other low-to-middle-income countries.

The present findings should be interpreted in the context of several potential limitations. The study was conducted with a convenient sampling technique which may affect the representativeness of the study sample. Although the Vietnamese translation of the MOS-SSS was validated, it had not been conducted on HIV/AIDS patients in Vietnam. Therefore, the results of concurrent validity are suggestive rather than affirmative.

In conclusion, findings from our study demonstrate that the Vietnamese translation of the MSPSS is a reliable and valid instrument in assessing the perceived social support for HIV/AIDS patients. This study also encourages the use of the MSPSS for HIV/AIDS population in other countries. Further studies on other populations are required to confirm our results.

## Data Availability

The data that support the findings of this study are available from the corresponding author, NLV, upon reasonable request.
